# American Medical Association—The Meeting at Milwaukee

**Published:** 1893-07

**Authors:** 


### AMERICAN MEDICAL ASSOCIATION—THE MEETING AT MILWAUKEE.

The meeting of the American Medical Association in Milwaukee was preceded by the meeting of the American Academy of Medicine, and that of the American Medical Editors Association. There was a full attendance of medical men from all sections of the country, excepting the South, which had rather a meagre representation, and Dr. Gaston was the only Southerner in attendance upon the American Academy of Medicine. The meetings of the Editors Association and of the American Medical Association, were addressed by Mr. Ernest Hart, the veteran Editor of the *British Medical Journal*, and his remarks, which have been published in the Journal of the A. M. A. and in the *News*, are well worthy of the consideration of the medical profession in the United States.

The different sections of the American Medical Association were generally well attended, but especially was the interest kept up in the sections of anatomy and surgery, of practice of medicine, and of obstetrics and diseases of women. There were many valuable papers read and discussed in each, and they will render the pages of the journal instructive to those who read them in the course of publication. The opening address, by Dr. Hunter McGuire, President of the Association, the annual address on General Medicine, by Dr. H. A. Hare, the annual address on Surgery, by Dr. Henry H. Mudd, and the address on State Medicine, by Dr. Walter Wyman, exhibited much thought and careful preparation in the several branches of investigation entered upon by the speakers.

The following officers for the ensuing year were elected: President, J. F. Hibbard; 1st vice-President, J. A. Wyeth; 2nd vice-President. I. N. Love. Trustees—J. B. Hamilton, L. S. McMurty, E. F. Ingalls, E. E. Montgomery. Council—

J. N. Quinby, J. McF. Gaston, A. F. Jonas and H. J. Murphy, editor of *Journal*, Dr. J. C. Culbertson.

The next meeting of the Association will be held at San Francisco, after a lapse of twenty-two years since a meeting was held at that place.

The master points in regard to changes in the Constitution and in the Code of Ethics, which had been under consideration by committees appointed at the Detroit meeting, were postponed until the next meeting of the Association.

The chairmen of the several sections were, by previous enactment, made members of the business committee, taking the place of the first on the list of those elected from each section at the last meeting.

In the surgical section, Dr. J. B. Roberts was elected chairman for the coming year and Dr F. W. McRae, Secretary, thus conferring a deserved compliment upon our colleague of this place, who has taken an active part in this section of the Association.

Dr. George M. Gould was elected President of the American Academy of Medicine, Dr. C. C. Bomburg, 1st vice-President, Dr. J. McFadden Gaston, 2nd vice-President. and Dr. Charles McIntire, Secretary and Treasurer.

Dr. C. H. Hughes, was chosen to preside over the next meeting of the American Editors Association.

The receptions extended to the members of the American Medical Association by the citizens of Milwaukee, constituted a most notable feature of the meeting.

Thc re-union dinner of the American Academy of Medicine, at the Plankington Hotel, on Saturday night, was a most enjoyable occasion. The toasts were interposed with singing, and the ladies joined heartily in this part of the performance.

A sumptuous banquet was served at the Phister Hotel for the Editors Association, at which the crowning feature was an address by Mr. Ernest Hart, on Medical Journalism.

